# Editorial: Omics-driven crop improvement for stress tolerance, Volume II

**DOI:** 10.3389/fpls.2024.1461217

**Published:** 2024-08-26

**Authors:** Yi Han, Weicong Qi, Jian Chen, Zhen Li, Xiaofeng Su, Freddy Kuok San Yeo

**Affiliations:** ^1^ National Engineering Laboratory of Crop Stress Resistance Breeding, School of Life Sciences, Anhui Agricultural University, Hefei, China; ^2^ Excellence and Innovation Center, Jiangsu Academy of Agricultural Sciences, Nanjing, China; ^3^ International Genome Center, Jiangsu University, Zhenjiang, China; ^4^ Vlaams Instituut voor Biotechnologie (VIB) Center for Plant Systems Biology, Department of Plant Biotechnology and Bioinformatics, Ghent University, Gent, Belgium; ^5^ Biotechnology Research Institute, Chinese Academy of Agricultural Sciences, Beijing, China; ^6^ Faculty of Resource Science and Technology, University of Malaysia Sarawak, Kota Samarahan, Sarawak, Malaysia

**Keywords:** omics, crops, abiotic and biotic stresses, breeding, stress tolerance

Crops are vulnerable to biotic and abiotic stresses that lead to reduced yields. Biotic stresses, such as fungi and pests, cause crops to rot and develop diseases. In contrast, abiotic stresses—such as high temperatures, salinity and mineral toxicity, and water shortages—irreversibly affect crops at different developmental stages, such as flowering, grain filling, and maturation through signal transduction, gene expression, and protein modifications. An increase of 1°C in the global average temperature is projected to significantly reduce crop yields. Excessive soil salinity tends to inhibit plant growth, hinder photosynthesis, and require metabolic adjustments. However, some crop types and species can tolerate modest levels of salinity without affecting their growth and yield. Drought is one of the most damaging abiotic stresses affecting severely the productivity of cereal crops. Rice struggles to survive in water-deficient fields, while maize is highly susceptible to drought. Therefore, improving crop stress tolerance is crucial for yield stability and healthy growth.

Advancements in omics technologies, such as genomics, transcriptomics, proteomics, and metabolomics, have significantly increased the feasibility and depth of biological research. These technologies offer a more comprehensive and holistic view of crops from different perspectives, which is critical for breeders. Additionally, the application of multiple omics effectively addresses bottlenecks that single omics technologies struggle to overcome. The advent of high-throughput, large-scale, and highly sensitive sequencing technologies, such as next-generation sequencing (NGS), has made genome sequencing more affordable and accessible. The integrated approach of genome-wide association studies (GWAS) and NGS technologies has provided significant opportunities for predicting stress-related genes. Transcriptomics focuses on the study of gene transcription and transcriptional regulation in a specific tissue or cell at specific developmental stages or conditions. Conversely, proteomics is the study of the composition and changes in protein content within cells, tissues, or organisms at various stages. These two fields reflect gene expression in the organism at two different molecular levels and are useful for gaining a deeper understanding of the intrinsic relationship between proteins and gene expression. They reveal gene expression and post-transcriptional regulatory states, providing more comprehensive information on the expression profile of the organism. Metabolomics, which is emerging after genomics and proteomics, significantly complements the understanding of cellular signaling, energy transfer, and intercellular communication after gene transcription and protein modification under genetic and environmental influences.

Currently, omics approaches play a crucial role in understanding crop stress resistance, disease resistance mechanisms, related-gene function research, germplasm resource identification, and assisted breeding. Recently, the application of omics strategies in crop stress tolerance research has significantly contributed to the advancement of the third “Green Revolution” in agriculture. Combining multiple omics approaches can reveal the molecular mechanisms underlying crop responses to stress and provide new strategies for developing stress-resistant varieties. Researchers have made significant progress in improving crop stress resistance using omics tools. Examples include enhancing stripe rust resistance in wheat (Lai et al.), necrotrophic bacterial disease resistance in solanum (Joshi et al.), drought tolerance in sugarcane (Li et al.), salinity stress resistance in cotton (Chen et al.), heat tolerance in grapes (Wu et al.), the response to grazing stress in *Phaeodactylum tricornutum* (Liu et al.) and antioxidant ability in postharvest crops (Wang et al.).

Stripe rust, caused by the fungus *Puccinia striiformis f.sp. tritici (Pst)* is a major threat to global wheat production. Lai et al. phenotyped the seedlings of 137 spring and 149 winter wheat varieties from Xinjiang, China, for resistance to six races of stripe rust. Subsequently, combining molecular markers, 10 stripe rust resistance genes that may correlate with the differences in resistance among wheat varieties were identified.

*Pectobacterium* species cause severe diseases, leading to rotting and wilting in cultivated potatoes, while wild potato relatives exhibit strong resistance. Studies have demonstrated that phytochemicals play a critical role in antivirulence traits. Therefore, Joshi et al. analyzed the metabolites in a population derived from a hybrid of *S. tuberosum* and *S. chacoense*. Approximately 30 metabolites, including alkaloids, terpenes, and several prenylated compounds, were found to be associated with resistance. Additionally, a QTL analysis was conducted to explore the genetic basis of antivirulence traits using recombinant inbred lines. Genetic mapping identified five QTLs associated with the inhibition of quorum sensing and two QTLs associated with the inhibition of protease activity. The metabolomics and QTL results demonstrated that quorum sensing inhibition and exo-protease inhibition could be efficient strategies for breeders to improve potato resistance to necrotrophic bacterial pathogens.

To identify drought resistance regulator genes and aid breeders in improving sugarcane (*Saccharum* spp.), Li et al. conducted a comparative transcriptomic study of two sugarcane cultivars with significantly different drought tolerance. The transcriptome results indicated that differential gene expression in various metabolic pathways, especially the photosynthesis pathway, may be the primary cause of the different drought tolerances in sugarcane varieties.

Cotton is vulnerable to abiotic stresses that often affect the production of fiber and cottonseed oil. A-galactosidases (AGALs), encoded by raffinose family oligosaccharide (RFO) catabolic genes, have been reported to be involved in plant stress tolerance. Chen et al. sequenced the whole genomes of *Gossypium hirsutum*, *Gossypium arboreum*, *Gossypium barbadense*, and *Gossypium raimondii*. Based on the sequencing results and genetic evidence, the *GhAGAL3* gene was shown to contribute to improved cotton tolerance to salinity stress by increasing the raffinose content.

With the increasing frequency of extreme weather events, high temperatures pose an unavoidable environmental threat to crops such as grapes, which are essential for fruit consumption and wine production. Wu et al. evaluated the heat tolerance of four grape varieties. Subsequently, RNA-seq was conducted on the four grape varieties under different temperature conditions. Based on bioinformatics analysis and differential expression comparisons, coexpression networks were constructed, identifying six genes associated with grape heat tolerance.

The quality of harvested crops is the basis for gaining economic benefits. Enzymatic browning reactions, triggered by oxidative stress, significantly compromise the quality of harvested crops. Therefore, a better understanding of the mechanism underlying browning is essential for the development of anti-browning treatments. Wang et al. unraveled enzymatic browning in crops by integrating omics strategies, including transcriptomic, proteomic, and metabolomic methods.

In conclusion, these Research Topics offer new insights into the application of omics tools for crop improvement in stress tolerance. Multi-dimensional omics studies have generated extensive datasets, revealing various molecular, physiological, and metabolic pathways related to abiotic and biotic stress tolerance, thereby opening new horizons for future investigations. The complex genome structure, variant epigenetic regulation and post-transcriptional modification and unpredictable environmental condition are still big challenges for the effective applications of omics strategies. Much more sensitive and higher resolution sequencing techniques, detective tools, and more scientific analysis methods are needed to advance research on polyploid crops.

